# Type 2 diabetes induced microbiome dysbiosis is associated with therapy resistance in pancreatic adenocarcinoma

**DOI:** 10.1186/s12934-020-01330-3

**Published:** 2020-03-24

**Authors:** Kousik Kesh, Roberto Mendez, Leila Abdelrahman, Santanu Banerjee, Sulagna Banerjee

**Affiliations:** 1 Department of Surgery, Miller School of Medicine, University of Miami, Miami, FL USA; 2 Sylvester Comprehensive Cancer Center, Miami, FL USA; 3 Miami Integrative Metabolomics Research Center, University of Miami, Miami, FL USA; 4 Department of Surgery, Miller School of Medicine, University of Miami, Biomedical Research Building, Suite 508, 1501, NW 10th Ave, Miami, FL 33156 USA; 5 Department of Surgery, Miller School of Medicine, University of Miami, Biomedical Research Building Suite 516, 1501, NW 10th Ave, Miami, FL 33156 USA

## Abstract

Resistance to therapy is one of the major factors that contribute to dismal survival statistics in pancreatic cancer. While there are many tumor intrinsic and tumor microenvironment driven factors that contribute to therapy resistance, whether pre-existing metabolic diseases like type 2 diabetes (T2D) contribute to this has remained understudied. It is well accepted that hyperglycemia associated with type 2 diabetes changes the gut microbiome. Further, hyperglycemia also enriches for a “stem-like” population within the tumor. In the current study, we observed that in a T2D mouse model, the microbiome changed significantly as the hyperglycemia developed in these animals. Our results further showed that, tumors implanted in the T2D mice responded poorly to gemcitabine/paclitaxel (Gem/Pac) standard of care compared to those in the control group. A metabolomic reconstruction of the WGS of the gut microbiota further revealed that an enrichment of bacterial population involved in drug metabolism in the T2D group. Additionally, we also observed an increase in the CD133+ tumor cells population in the T2D model. These observations indicated that in an animal model for T2D, microbial dysbiosis is associated with increased resistance to chemotherapeutic compounds.

## Introduction

Pancreatic cancer is the 3rd most prevalent cause of cancer related deaths in United states alone, with over 55,000 patients being diagnosed in 2019 alone and nearly as many succumbing to it. Late detection, lack of effective therapy and poor understanding of pancreatic cancer systemically contributes to its poor survival statistics. Alarmingly, this disease has been projected to emerge as the 2nd most common of cancer related deaths in United States by 2030. While there have been extensive studies at the tumor intrinsic as well as around the contributions of the microenvironment towards the aggressive biology of pancreatic tumors, not a lot of research has been focused on the contributions of systemic factors in its poor outcome.

Obesity and high caloric intake linked co-morbidities like type 2 diabetes (T2D) have been attributed as being risk factors for a number of cancers including pancreatic cancer [1, 2]. Apart from being considered as one of the risk factors for pancreatic cancer, late onset of diabetes is now being evaluated as a potential early biomarker for pancreatic cancer [3–5]. Studies have shown that pancreatic cancer patients with T2D have poor survival statistics [6]. Additionally, patients with T2D often show severe hyperglycemia while undergoing anti-neoplastic chemotherapy [7]. Furthermore, T2D has been associated with an increased risk of chemotoxicity and heightened mortality rate in cancer patients [8, 9]. Studies show that organ dysfunction like nephropathy, vasculopathy and polyneuropathy associated with T2D impact the dosages of chemotherapy tolerated by the patients and often contribute to risk of toxicity [10]. Thus, poor survival in cancer patients with T2D is due to less efficacy of chemotherapy. The mechanism by which T2D and hyperglycemia can induce chemoresistance has remained elusive. It is possible that increase glucose contributes to the enrichment of “stem” like population that is treatment refractory, and hence responsible for therapy resistance [11]. Studies from our laboratory have identified a CD133+ population within pancreatic cancer cells that exhibit extreme resistance to all known chemotherapy [12–15]. However, whether this population is enriched under T2D was not evaluated.

Studies on gut microbiome has shown that lifestyle factors as well as diet has a huge effect on the microbial flora of the gut [16–19]. Further, modulation of gut microbiome has been seen to contribute to effects of intensive insulin therapy in mice on high fat diet [20]. In another study, abnormal gut microbiota was reported to contribute to development of diabetes in Db/Db mice [21]. Recent studies indicate that microbiome and microbial dysbiosis plays a role in not only the onset of disease but also in its outcome. In colorectal cancer, *Fusobacterium* has been reported to promote therapy resistance [22]. Certain intra-tumoral bacteria have also been shown to elicit chemoresistance by metabolizing anti-cancerous agents [23, 24]. In pancreatic cancer, studies on altered gut microbiome have been relatively recent. Microbial dysbiosis has been observed to be associated with pancreatic tumor progression [25, 26]. Modulation of microbiome has been shown to affect response to anti-PD1 therapy in this disease as well [27]. However, most of the studies in pancreatic cancer and microbiome have remained focused on immune modulation.

Previous studies from our laboratory has shown that whole genome sequencing (WGS) of the gut microbiome provide a deeper coverage of the DNA sequences compared to bacterial 16S pyrosequencing. Additionally, this method also enables us to perform metabolomic reconstruction analysis that can determine the groups of bacterial population that have been enriched as a result of disease of as a response to therapy [25]. The reconstruction studies can then be used to predict the microbial metabolites that can influence a host or therapy response.

In the current study, we observed that in a T2D mouse model, the microbiome changed significantly as the hyperglycemia developed in these animals. Our results further showed that, tumors implanted in the T2D mice responded poorly to standard of care chemotherapy in pancreatic cancer, gemcitabine/paclitaxel (Gem/Pac) compared to those in the control group. A metabolomic reconstruction of the WGS of the gut microbiota further revealed that an enrichment of bacterial population involved in drug metabolism in the T2D group. Additionally, we also observed an increase in the CD133+ population in the T2D model. These observations indicated that in an animal model for T2D, microbial dysbiosis is associated with increased resistance to chemotherapeutic compounds.

## Methods

### Experimental animals

C57BL/6J mice were obtained from the Jackson Laboratory (Bar Harbor, Maine, USA). All mice were male and 4–6 weeks old. Food and tap water were available ad libitum. All mice were housed four mice per cages and maintained on a 12-h light/dark cycle, in a constant temperature (72 ± 1 °F) and 50% humidity. All procedures were conducted according to the protocols approved by the University of Miami Institutional Animal Care and Use Committee (IACUC).

### Animal model for type 2 diabetes

Thirty-two male C57BL/6J mice were first randomly divided into two groups (WT group, and T2D group). Mice in the T2D group were given an intraperitoneal injection of sterile citrate buffer containing streptozotocin (25 mg/kg, Sigma Aldrich, St. Louis, MO, USA), while mice in the WT group were injected intraperitoneally with the same volume of citrate buffer. After continuous administration of streptozotocin for 5 days, the T2D group was fed a high-fat diet (42% Cal from fat, TD.88137, ENVIGO) for 4 weeks to establish a model of type 2 diabetes. The WT group was given adjusted control diet (4% Cal from fat, TD. 08405, ENVIGO).

### Tumor implantation

5000 number of pancreatic cancer cells (KPC001) were implanted in both groups of mice after 4 weeks of diet. After 2 weeks of tumor implantation when subcutaneous pancreatic cancer model was established, the two groups of mice were each further randomly divided into two subgroups (WT and WT + GP; T2D and T2D + GP). The WT + GP and T2D + GP groups received intraperitoneal injections of 100 mg/kg of gemcitabine and 10 mg/kg of paclitaxel twice in a week for consecutive 4 weeks, while the WT and T2D groups were only receive equal volume of saline.

### Fecal matter collection and DNA isolation

Fecal samples were collected in different time point to understand the effect of several sequential treatment in gut microbiota. Fecal samples collection was performed after 1, 4, 6 and 10th weeks’ after 1st injection of streptozotocin. Samples were collected in a sterile Eppendorf tube inside a biosafety cabinet with sterile forceps. Each group consisting eight animals were randomized (group wise) to nullify cage-effect in microbiome studies among the groups. After 10 weeks, all animals were sacrificed according to protocols approved by University of Miami Animal Care Committee. Part of the tumor sample were flash frozen in liquid nitrogen, while the rest were formalin fixed for paraffin embedding and histochemical analysis. Blood was collected by cardiac puncture prior to euthanizing the animals. Serum samples were stored for biochemical analysis. DNA from the murine fecal samples was isolated using the Power Soil DNA Isolation Kit (Qiagen) according to manufacturer’s instructions. All samples were quantified using the Qubit^®^ Quant-iT dsDNA High-Sensitivity Kit (Invitrogen, Life Technologies, Grand Island, NY) to ensure that they met minimum concentration and mass of DNA and were submitted to University of Minnesota Genomics Center for Whole Genome Sequencing.

### Metagenomic sequencing and microbiome analysis

Shotgun metagenomic library was constructed from fecal DNA with the Nextera DNA sample preparation kit (Illumina, San Diego, CA), as per manufacturer’s specification. Barcoding indices were inserted using Nextera indexing kit (Illumina). Products were purified using Agencourt AMpure XP kit (Beckman Coulter, Brea, CA) and pooled for sequencing. Samples were sequenced using MiSeq reagent kit V2 (Illumina) in a HiSeq2500 sequencer.

Raw sequences were sorted using assigned barcodes and cleaned up before analysis (barcodes removed and sequences above a quality score, Q ≥ 30 taken forward for analyses). For assembly and annotation of sequences, MetAMOS [28] pipeline or Partek Flow software (Partek^®^ Flow^®^, Partek Inc., St. Louis, MO) were used. These softwares provide powerful tools to filter unique hits between human and mouse-specific genes versus microbial signatures. Alpha and Beta diversity calculations were done using embedded programs within the metagenomic pipeline, or using Stata15 (StataCorp LLC, College Station, TX) or EXPLICET software [29].

Functional profiling was performed using HUMAnN2-0.11.1 [30] with Uniref50 database to implement KEGG orthologies.

### Blood glucose and cholesterol measurement

Blood samples were collected by retro-orbital sinus puncture via the medial canthus of the eye using clean 44.7-μL heparinized micro hematocrit tubes. No anesthesia was used at the time of the blood sampling, to avoid unequal variations between animals and avoid the effects of anesthesia on the blood glucose levels. Mice blood glucose was measured using true track blood glucose meter and strips (Trivida health). Measurement of total cholesterol from mice serum were performed using cholesterol assay kit (Abcam) according to the manufacturer’s instructions.

### Histology and immunofluorescence staining

Tumor from all groups of mice were sectioned for histological studies. The tissue samples were fixed in 10% formalin and embedded in paraffin. The sections (5 μm) were cut using microtome, stained with hematoxylin and eosin, and slides were assessed using microscope (Leica microsystems, Germany) using at original magnification 10× and processed in Adobe Photoshop. For the immunofluorescence study, paraffin embedded sections were deparaffinized with xylene followed by rehydration with descending alcohol series. Slides were steamed with Reveal Decloaker (Biocare Medical), to minimize background staining, Sniper Universal Blocking Sera (Biocare Medical) was used throughout the protocol. Primary antibodies for Ki67 (Invitrogen), cd133 (Invitrogen) were diluted according to the manufacturer’s instruction and incubated overnight at 4 °C. Subsequently, Alexa Fluor-conjugated secondary antibody (Invitrogen) were used for visualizing. Slides were counterstained with DAPI and observed in a Leica fluorescent microscope. Negative control slides were used to discriminate nonspecific staining.

### Sirius red staining and measurements

Tissue sections were deparaffinized and hydrated in a descending order of alcohol solution, followed by PBS washing. Collagen staining were performed using picrosirius red staining solution (Chondrex Inc). The sections were washed with acidified water and dehydrated using absolute alcohol followed by mount in a resinous medium. The Sirius red-stained area was quantified using ImageJ software by selecting stained fibers in randomly selected five fields at a magnification of 10× under a light microscope.

### Quantitative real-time PCR assay

Quantitative real-time PCR for CD133, SOX2, OCT-4 and Nanog1 were performed using primers from Qiagen (QuantiTect primer assay). RNA extraction was performed using Trizol (Invitrogen), according to the manufacturer’s instructions. The High Capacity cDNA Reverse Transcription Kit (Applied Biosystems) was used to convert 2 μg of RNA to cDNA. Gene expression was analyzed through qRT-PCR using LightCycler 480 System (Roche) and SYBR Green (Qiagen). The 18s ribosomal RNA expression was used to normalize the results obtained in different conditions (18S QuantiTect Primer Assay; Qiagen). Primers used in this study were obtained from (Qiagen, Valencia CA).

### Statistical analysis

Data were presented as the mean ± SD. Statistical analyses were performed using SPSS software, version 18.0. Differences between two groups were analyzed by Student’s *t* test. *p *< 0.05 was considered statistically significant. Most statistical functions for microbiome and metabolome were embedded within MetAMOS [28] pipeline or Partek Flow software (Partek^®^ Flow^®^, Partek Inc., St. Louis, MO). Output files from microbial sequence analysis and predictive metabolomics were further subjected to groupwise comparison. Depending on the analysis (as mentioned in respective figure legends), test of significance was either Mann–Whitney U test (Graphpad Prism), one-way ANOVA or 2-tailed t-test with false discovery rate (FDR) correction (using Bonferroni or Benjamin–Hochberg correction). The FDR threshold was set at 0.1 and p < 0.05 was considered to be significant.

### Ethics statement

All animal studies were performed according to the protocols approved by IACUC at University of Miami, USA in accordance with the principles of the Declaration of Helsinki. All authors had access to all data and have reviewed and approved the final manuscript.

### Availability of data and materials

The dataset(s) supporting the conclusions of this article is(are) available in the ArrayExpress repository, Identifier: E-MTAB-8739, https://www.ebi.ac.uk/arrayexpress/.

## Results

### Mice with metabolic syndrome show resistance to therapy

**Fig. 1:**
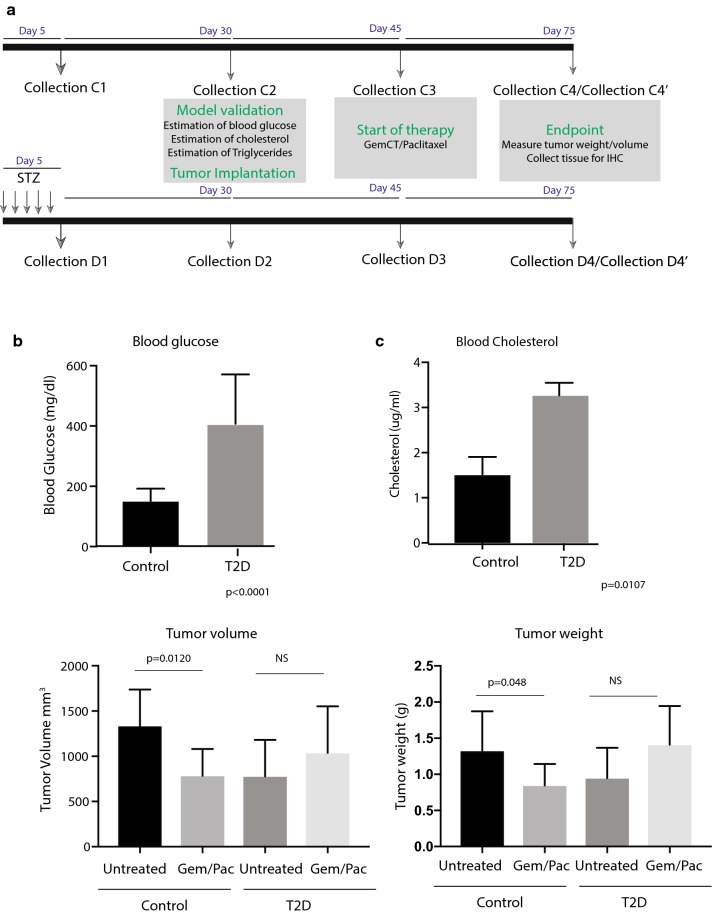
T2D contributes to therapy resistance in PDAC. Schematic diagram of the experimental setup. Arrows indicate Streptozotocin (STZ) administration and fecal matter collection. C1, C2, C3, C4 refer to control groups that do not receive STZ or high fat diet D1, D2, D3, D4 refer to animals receiving STZ and put on high fat diet. C4 and D4 groups were further split into C4′ and D4′ to indicate groups that received Gem/Pac therapy (**a**). Blood glucose (**b**) and blood cholesterol (**c**) was measured at the end of 1 month to validate hyperglycemia and T2D onset in animals. After tumor implantation and 1 month of Gem/Pac, T2D group animals did not show any significant change in tumor volume (**e**) or weight (**f**), while the control tumors responded to the standard of care. *Indicates p value = 0.05

To develop a type 2 diabetes (T2D) model, we injected streptozotocin 5 days prior to putting animals in a high fat diet (details in method, Fig. 1a, Table 1). After 30 days of high fat diet (42% calories from fat), the animals were tested for changes in body weight, blood glucose, blood triglyceride and cholesterol levels. Our results showed that animals receiving streptozotocin (STZ) and high fat diet did not show a significant change in body weight (Additional file 1: Figure S1A). However, these animals had 2.5-fold increase in their blood glucose level (Fig. 1b) Along with this, the cholesterol level in these animals were significantly higher than the one in the control group (Fig. 1c). This indicated that our streptozotocin + high fat diet mimicked the type 2 diabetes model (T2D) in these animals and they had glucose and lipid profiles similar to that observed in patients. We next implanted murine pancreatic cancer cells KPC001 (isolated from primary KPC tumors) subcutaneously in these animals. There was no observed difference in the tumor take rate between the two groups (Additional file 1: Figure S1B).

**Table 1:** Fecal sample collection scheme

Name of collection	Day of collection	Event	Event affecting microbiome change
C1	5	No treatment	Control vs Onset of T2D
D1	5	After 5 STZ injections	
C2	30	Adjusted control diet, tumor implanted	Control vs established T2D
D2	30	High fat diet, tumor implanted	
C3	45	Tumor bearing control animals randomized into untreated and Gem/Pac treatment group	Effect of tumor on microbial change in control vs. diabetes animal
D3	45	Tumor bearing T2D animals randomized into untreated and Gem/Pac treatment group	
C4	75	Control animals in untreated arm sacrificed; tissues and fecal matter collected	Effect of therapy on microbial change in all groups
D4	75	T2D animals in untreated arm sacrificed; tissues and fecal matter collected	
C4′	75	Control animals in Gem/Pac arm sacrificed; tissues and fecal matter collected	
D4′	75	T2D animals in Gem/Pac arm sacrificed; tissues and fecal matter collected	

To study if the animals with T2D showed differential response to standard therapy in pancreatic cancer, we next treated the control group and the T2D group with standard of care treatment gemcitabine/paclitaxel (Gem/Pac) at a dose of 100 mg Gem/10 mg Pac. Our results showed that while the animals in the control group responded to Gem/Pac treatment, the T2D mice were resistant to it (Fig. 1d, e).

### Tumor bearing animals with T2D show distinct histological features

**Fig. 2:**
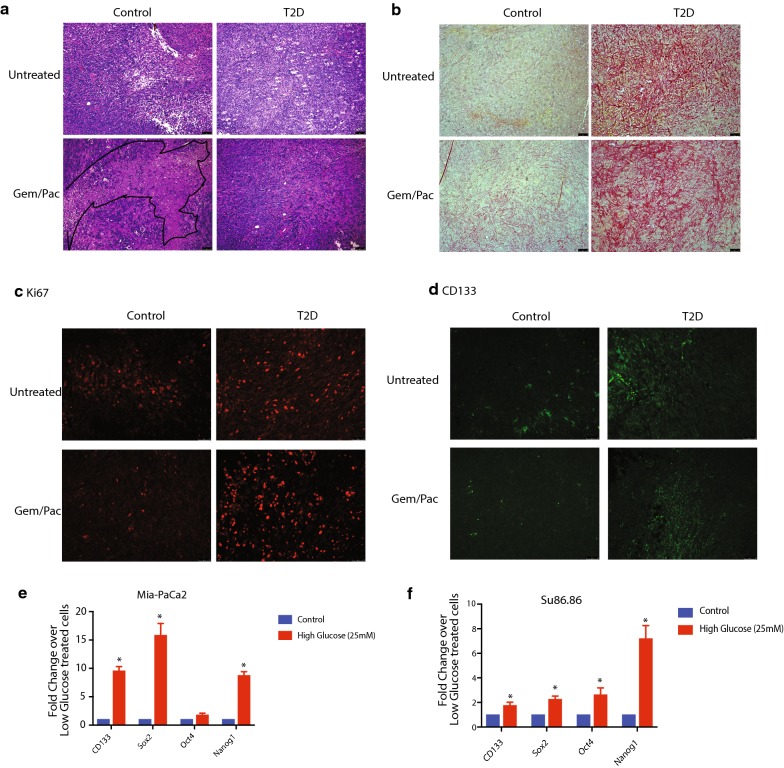
T2D mice had tumors with increased fibrosis and stem-like population: H&E section of the pancreatic tumors (**a**), Picrosirius red staining showing deposition of collagen in T2D mice that were unchanged upon treatment with Gem/Pac (**b**). T2D tumors showed increased proliferation (**c**) and CD133+ (**d**) staining that remained unchanged with therapy. In our in vitro experiments, conditions of high glucose increased expression of stemness genes in MIA-PACA2 (**e**) and Su86.86 (**f**) cell lines

In order to determine how diabetic animals mediated therapy resistance in this model, we next performed a histological analysis of the tumor. H&E stained slides showed presence of hyperproliferative cells typically observed in subcutaneous tumors in both control and T2D animals. However, there were large areas of necrosis and cell death observed in the Gem/Pac treated animals in the control group (region marked in black), while the group in T2D animals showed no observable difference in histology (Fig. 2a). Our analysis also showed that the tumor bearing T2D animals had increased deposition of collagen compared to the control animals. Treatment with paclitaxel, did not affect the collagen in these tumors (Fig. 2b). Further, tumors in T2D animals were more proliferative than those in the control animals as seen by Ki-67 staining (Fig. 2c). As we observed in Fig. 1, treatment with Gem/Pac, decreased Ki-67 cells in tumors of control animals but did not affect those in the T2D animals (Fig. 2c, Additional file 2: Figure S2A). Since resistance to chemotherapy is associated with an increased in cancer stem cell population, we next stained the tumors with anti-CD133 antibody, that has been previously demonstrated as a cancer stem cell marker and has been associated with therapy resistance. Tumor bearing animals in T2D group showed more CD133 staining compared to those in the control group (Fig. 2d, Additional file 2: Figure S2B). Interestingly, increasing glucose in the culture media also showed an increase in the CD133 expression as well as expression of other cancer stem cell markers like Sox2, Oct4 and Nanog in our pancreatic cancer cell lines MIA-PaCa2 and Su86.86 (Fig. 2e, f).

### T2D model showed microbial dysbiosis

**Fig. 3:**
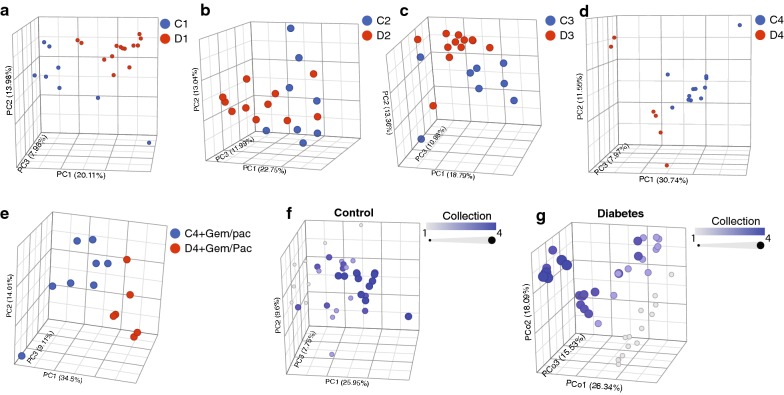
T2D animals showed distinct change in gut microbiome: Bray–Curtis principal component clusters indicating a significant change in the microbial population between control and T2D groups in collection 1 (**a**), collection 2 (**b**), collection 3 (**c**) and collection (**d**). Distinct clustering was observed between the C4′ and D4′ groups receiving treatments as well (**e**). PCoA plots within control group showed no distinct clustering (**f**), while in T2D group separate collections tended to group separately (**g**)

Since change in physiology is closely linked with microbial dysbiosis, we next analyzed the change in the gut microbiome during model development. Figure 1a shows the events from each collection in the control and diabetes model. A comparison of gut microbiome between the control (C1) and streptozotocin (STZ) treated (D1) animals showed a well separated Bray–Curtis principal component clusters indicating a significant change in the microbial population (Fig. 3a). These differences are maintained after 30 days (Fig. 3b), 45 days (Fig. 3c) and 75 days (Fig. 3d) of STZ treatment. Cohort of animals introduced to gemcitabine/paclitaxel treatment beyond day 45, i.e. groups C4′ and D4′ for controls and diabetes respectively, also exhibit distinct clustering for their microbial composition (Fig. 3e). It was also evident from the analysis of all four collections from control animals that over the course of 75 days that their microbiome has minimal differences over time (Fig. 3f). While there are differences between the first collection and the last (which is expected due to non-STZ treatments), no distinct clustering was observed within controls. In comparison, diabetic mice showed distinct clusters for all four collections with minor overlap for collections 2 and 3 (30 and 45 days post STZ; Fig. 3g).

**Fig. 4:**
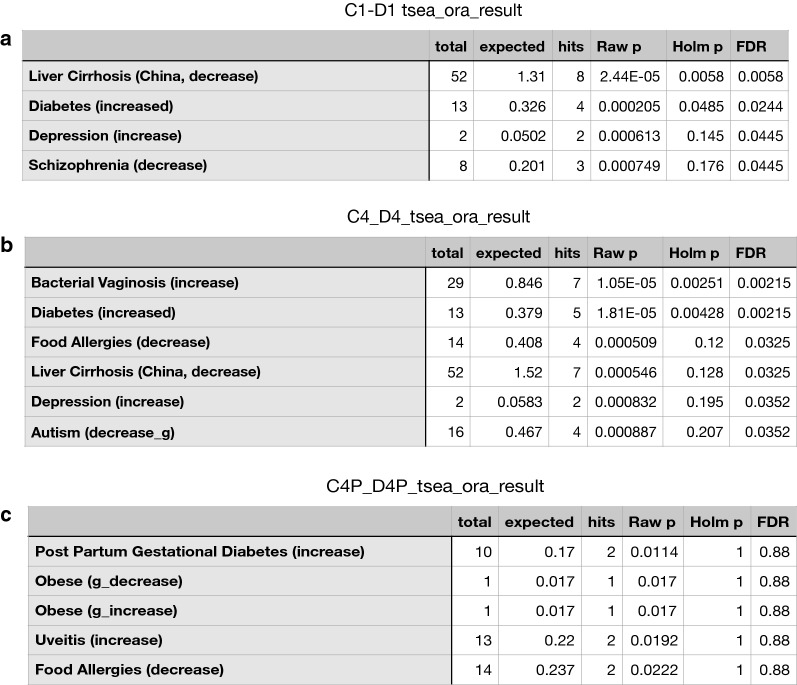
Taxon set enrichment analysis (TSEA): Significantly correlated human diseases with known composition of altering flora between in C1–D1 (**a**) and C4–D4 sets (**b**) showing strong correlation with increased diabetes in humans. Chemotherapeutic groups C4′ and D4′ microbiome did not exhibit any significant correlation with any human disease (first five hits shown for representation (**c**)

**Fig. 5:**
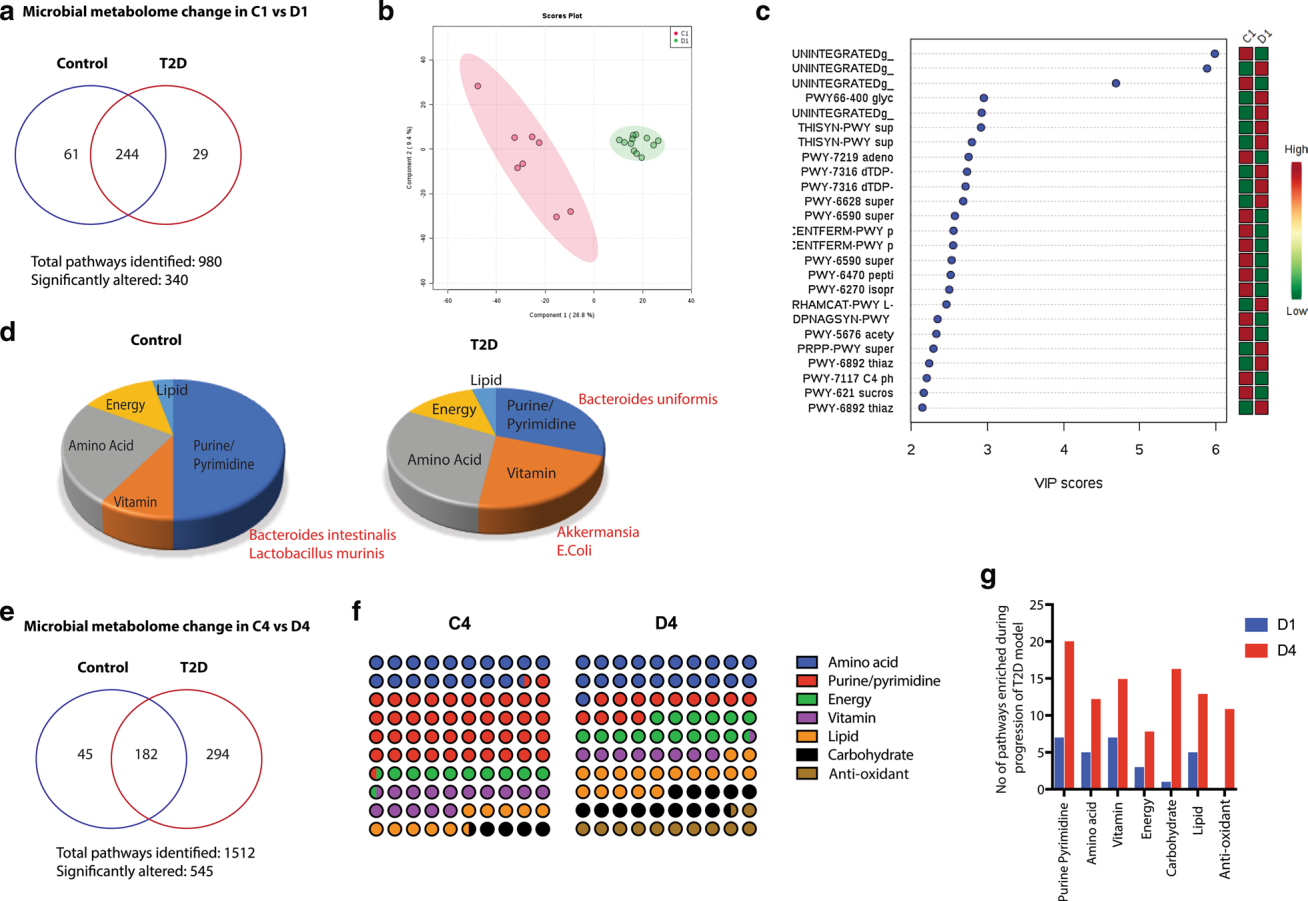
T2D showed altered microbial metabolome: Metabolomic reconstruction using humaN2 pipeline was performed to determine the microbial metabolome. Venn diagram representing enriched metabolites in C1 vs D1 (**a**). A partial least squares-discriminant analysis (PLS-DA) plot shows distinct clustering of the overall pathway composition within the two groups, implying major differences between the metabolic profiles of the two groups (**b**). A Variable Importance in Projection (VIP) analysis on the first principle component of PLS-DA plot identifying 22 named pathways significantly changing between C1 and D1 (**c**). Enrichment of metabolic groups in control and T2D groups visualized by pie charts (**d**). Venn diagram showing altered metabolites in C4 vs D4 (**e**). Distinct enrichment of bacteria that metabolize anti-oxidants in the D4 which was absent in C4 observed in parts-of-whole analysis (**f**). An enrichment of almost all the general metabolic pathways was observed in D4 when compared to D1 (**g**)

Next, we performed Taxon Set Enrichment Analysis (TSEA) of all significantly changing genera using the TSEA online tool hosted at MicrobiomeAnalyst (https://www.microbiomeanalyst.ca/) and parsed them across 239 taxon sets associated with human diseases (host-intrinsic). Figure 5 represents significantly correlating human diseases with known composition of altering flora between the group pairs. Both C1–D1 and C4–D4 sets (Fig. 4a, b respectively) show strong correlation with increased diabetes in humans. The 75-day collection (C4–D4) additionally exhibited strong correlation with inflammatory and autoimmune disorders. Chemotherapeutic groups C4′ and D4′ microbiome did not exhibit any significant correlation with any human disease (first five hits shown for representation; Fig. 4c).

### T2D model showed altered microbial metabolome

Since microbial metabolome contributes significantly to both host physiology as well as pathology, we next performed a metabolic reconstruction using our WGS obtained from the fecal samples of control and T2D animals using the HUMAnN2 pipeline [30, 31]. Three distinct datasets were obtained: (a) significant pathways in C1 (control animals prior to any treatment or diet) vs D1 (Animals after 5 injections of streptozotocin to induce T2D), (b) significant pathways in C4 (end of study in control animals) vs D4 (end of study animals in the T2D group) and (c) C4′ (end of study control animals that received Gem/Pac) vs D4′ (end of study T2D animals that received Gem/Pac). The resulting pathways were classified and curated manually into: (i) Microbial population enriched for pathways that were only present in the control animals (C1, C4 and C4′) and (ii) those that were only present in T2D group (D1, D4, D4′).

Our data showed that at the beginning of the study (C1 vs. D1), a total of 340 out of 980 pathways were significantly altered within the microbial compartment. Among these 61 metabolic pathways were enriched specifically in Control group and completely absent in the T2D group. Similarly, 29 pathways were enriched in the T2D groups, while being absent in the Control group (Fig. 5a and Additional file 3: Figure S3). A partial least squares-discriminant analysis (PLS-DA) plot shows distinct clustering of the overall pathway composition within the two groups, implying major differences between the metabolic profiles of the two groups (Fig. 5b). A Variable Importance in Projection (VIP) analysis on the first principle component of PLS-DA plot identified 22 named pathways significantly changing between the two groups (Fig. 5c), with a MetaCyc rendition provided in Additional file 4: Figure S4. In C1 vs D1 dataset, among the 244 metabolic pathways that were deregulated upon treatment with STZ, those contributing to amino acid metabolism were among the most deregulated (28% of the dataset), followed by purine/pyrimidine biosynthesis (22% of the dataset), deregulated energy metabolism pathways (14%) and vitamin metabolism (10%) pathways. Deeper analysis showed that upon streptozotocin treatment, i.e. in the D1 set, there was an enrichment of vitamin metabolizing bacteria. Interestingly, all the vitamin metabolism pathways that were active in *Akkermansia* and *E. coli* were upregulated in the STZ treated animals (Fig. 5d). The metabolomic reconstruction of the fecal bacteria in the control group showed that major metabolic pathways that were enriched belonged to the purine/pyrimidine metabolic pathways. We have observed this in our earlier studies as well [25]. Analysis of the metabolic pathways that were specifically absent in the control animals showed that there were relatively lesser pathways involved the purine/pyrimidine metabolism (~ 30%). Interestingly, the main bacterial population present in the control group were *Bacteroides intestinalis* and *Lactobacillus murinus*. These contributed to the purine/pyrimidine metabolism as well as vitamin metabolism. In the T2D group however, we observed an enrichment of *Bacteroides uniformis*, a bacterial species that was absent in the control samples (Fig. 5d).

**Fig. 6:**
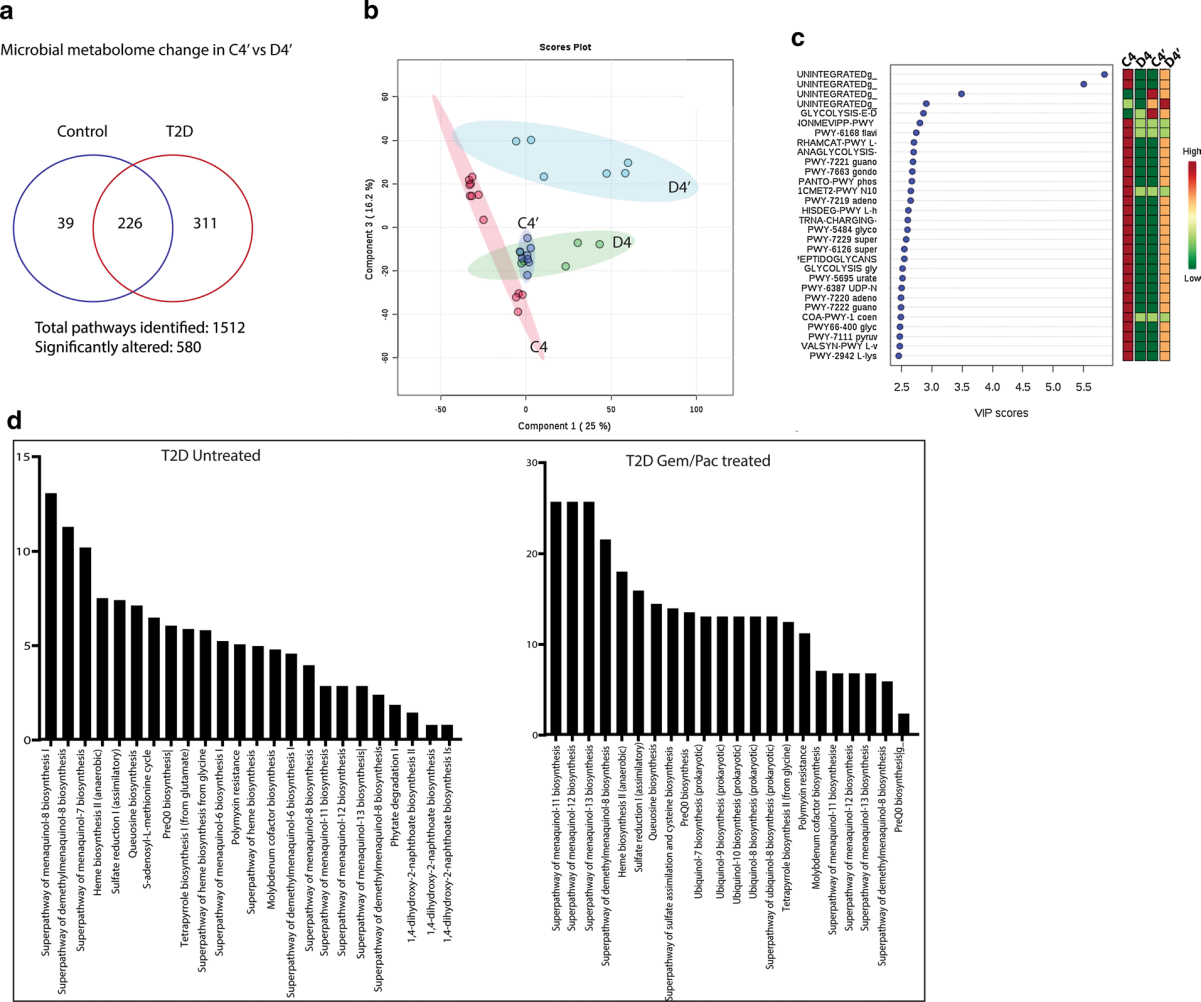
Microbial metabolome changes in chemotherapy treatment groups: Venn diagram representing changes in microbial metabolic pathways in chemotherapy treated groups (**a**). Combined PLS-DA analysis of C4, D4, C4′ and D4′ datasets shows distinct clustering of all four groups (**b**) in which only C4 and D4′ show well separated clusters, while D4 and C4′ show similar metabolic landscape within the gut microbiome. VIP analysis across the first principal component for all four groups show distinct down-regulation for D4, C4′ and D4′, compared to C4 controls (**c**). List of bacterial pathways that are deregulated in T2D (i.e. D4) and T2D group treated with chemotherapy or D4′ (**d**)

In C4 vs D4 dataset (75 days post STZ treatment in the D group), we observed a total of 1512 metabolic pathways of which 545 were significantly altered (Fig. 5e). Interestingly, in the C4 vs D4 dataset, we observed a drastic change in the microbial metabolome in the T2D group (D4) compared to C4 (Fig. 5f). There was a distinct enrichment of bacteria that metabolize anti-oxidants in the D4 which was absent in C4. The bacterial metabolic pathways also changed during progression of the T2D model. We observed an enrichment of almost all the general metabolic pathways in D4 when compared to D1. The major bacterial species enriched in D1 was *Bacterial uniformis*, while Enterobacter and specifically Enterobacter cloacae appeared to be enriched in D4 (Fig. 6g). None of these bacterial population were observed in the control samples (C1 and C4). Out of the 545 significantly altered metabolic pathways, 45 were only enriched in control and 294 were only enriched in T2D model.

In the chemotherapy treatment groups C4′ and D4′, 580 out of the 1512 pathways detected were significantly altered (Fig. 6a). In this dataset, 39 were enriched only in the control (C4′ set) while 311 were enriched only in the T2D set (D4′ set). Combined PLS-DA analysis of C4, D4, C4′ and D4′ datasets shows distinct clustering of all four groups (Fig. 6b). However, only C4 and D4′ show well separated clusters, while D4 and C4′ show similar metabolic landscape within the gut microbiome. A VIP analysis across the first principal component for all four groups returned a list of 26 named pathways contributing to the differences seen in the PLS-DA plot (Fig. 6c). The adjacent heatmap shows that at least for these pathways, there is a distinct down-regulation for D4, C4′ and D4′, compared to C4 controls (except glycolytic and non-mevalonate isoprene biosynthetic pathways). Additionally, D4′ and C4′ groups show a very similar profile for these pathways. A MetaCyc rendition of these pathways is presented in Additional file 5: Figure S5. Overall comparison of the relative enrichment of the microbial metabolites during the course of tumor development, we observed that carbohydrate and lipid metabolizing bacteria tended to be enriched in this model. 10% of carbohydrate and 11% of lipid metabolizing bacteria were enriched at the onset of the model, while at the 4th collection (end of experiment), there was an enrichment of 17% carbohydrate and lipid metabolizing bacteria. Since gut bacteria plays an integral part in metabolism, this further validated our T2D model.

Further analysis revealed an enrichment of oxidative stress “protectant” metabolites in the altered drug metabolism pathways in the D4 and D4′ group (Fig. 6d). These compounds like menaquinol and queuosine are microbial metabolites that act as anti-oxidants and offer cell protection from chemotherapy induced accumulation of reactive oxygen species [32]. It is possible that enrichment of this pathway in the T2D group results in their therapy resistant nature. Interestingly, the number of bacteria participating in each enriched pathway was more in the treatment group (D4′) compared to untreated (D4).

## Discussion

Cancer patients with diabetes often pose critical challenges to clinicians making therapy decisions. One of the major challenges is the poor response to the chemotherapeutic drugs. Recent studies have shown that high blood sugar levels can lead to oxaliplatin resistance in patients with Stage III colorectal cancer [33, 34]. Similarly, in breast cancer, hyperglycemia induced resistance was observed in the ER+ types [35]. In glioblastoma, a negative impact on elevated blood sugar on overall survival of patients was noted [36]. In prostate cancer, hyperglycemia decreased docetaxel induced apoptosis, thus showing a poor response to the drug [37]. Similarly, reversal of hyperglycemic conditions with anti-diabetic drugs like metformin have been observed to sensitize cancers to chemotherapy [38].

Interestingly, in pancreatic cancer, where diabetes is considered to be one of the risk factors, there is no studies on the how hyperglycemia affects the response to standard therapy. Standard of care in pancreatic cancer is typically gemcitabine. In 2013, since nab-paclitaxel became approved, gemcitabine–paclitaxel (Gem/Pac) combination therapy is one of the most used chemotherapy combination. Our study shows that hyperglycemia induces resistance to this therapy combination in pancreatic cancer cells as well (Fig. 1). While animals that have a well-regulated blood glucose respond to the Gem/Pac therapy, animals that were induced to be T2D, failed to respond. Additionally, the tumors in the T2D group showed extensive desmoplasia and collagen deposition, hyperproliferative tumor and increased presence of CD133+ cells (Fig. 2). Previous publications from our laboratory have identified CD133 as a marker of pancreatic cancer stem cells, that are extremely resistant to any therapy [12, 15]. These observations confirmed that hyperglycemia contributed to an aggressive tumor by affecting both its microenvironment as well as by enriching for cancer stem cells.

Like most disease, gut microbiota has been associated with diabetes [39]. Recent studies have shown that a gut microbial dysbiosis is instrumental in not only disease types but also their pathology. However, most studies are still at the correlational and whether the dysbiotic microbiome actually drives the disease process is not known. In our studies, the gut microbiome showed changes as early as 5 days of streptozotocin injections (C1 vs D1 in Fig. 3a). By the time the animals developed hyperglycemia (1 month of high fat diet), the microbiome was completely different from the control group (C2 vs D2 in Fig. 3b). Similarly, when compared within the T2D group (D1–D4), the change in microbial population was distinctive as the disease developed as observed in separate clusters of PCoA plots in Fig. 3g, while the microbial population remained almost unchanged in control group (Fig. 3f). Taxon Set Enrichment analysis or TSEA is used to identify microbes that share same phenotypical traits, association with host lifestyle. These include diet composition, body mass index (BMI), oxygen requirement etc. Thus, there is an enrichment of microbes associated with developmental, physiological or disease conditions that are similar [40]. Our TSEA of all significantly changing genera within the control and T2D groups showed that both C1–D1 and C4–D4 sets (Fig. 5) had strong correlation with increased diabetes in humans. This further validated our model and microbial changes associated with T2D. Next, we looked into the significantly changing bacterial genera within the individual groups. The bacterial groups were analyzed pairwise by sorting the list according to significantly changing genera (p < 0.05; FDR adjusted). Between C1 and D1 (control vs. diabetic), a total of 108 genera varied significantly. Additional file 6: Figure S6A represents the top 50 genera, where most showed a net increase in relative abundance, while *Cytophaga*, *Ruminoclostridium*, *Leuconostoc* and *Staphylococcus* were the only genera with net reduction in relative abundance. After 75 days of STZ treatment (C4 and D4), a total of 192 genera were found to be significantly altered and top 50 genera are represented in Additional file 6: Figure S6B. Interestingly, only 11 out of 50 genera were common within the two collection when compared pairwise between age-matched control and diabetic mice (between Additional file 6: Figure S6A and B). Only one genus, *Leuconostoc* showed a reduction in relative abundance. Upon gemcitabine/paclitaxel treatment (C4′ and D4′), 277 genera showed significant changes in relative abundance. All within the top 50 changing genera showed a net increase in relative abundance and none (except *Mycobacterium*) were common between treatment (C4′, D4′) and non-treatment (C4, D4) groups (Additional file 6: Figure S6C).

To understand if the change in the gut microbiome was associated with the resistance of the pancreatic tumors to chemotherapy, we next analyzed the microbial genome by HUMANn2. Our analysis showed that in the T2D group, the bacterial population responsible for synthesis of anti-oxidants was enriched. We specifically identified an enrichment of menaquinol synthesis. Menaquinol is a microbial metabolite that act as anti-oxidants and offer cell protection from chemotherapy induced accumulation of reactive oxygen species [32]. It is possible that enrichment of this group of bacteria in the T2D group confers chemoresistance to the tumors. While in a normal cell, presence of anti-oxidants is considered healthy, in a cancer cell this mechanism can protect from oxidative stress and thus lead to resistance. In a comprehensive study by Qin et al., Type 2 Diabetes microbiome was observed to be enriched for *Akkermansia muciniphila* and Bacteroides intestinalis [39]. In fact, *Akkermansia muciniphila* is currently being considered as a biomarker for glucose intolerance [41]. Our streptozotocin-high fat diet T2D model showed similar enrichment of bacterial species. Additionally, an enrichment of carbohydrate and lipid metabolizing bacteria observed in T2D group in Fig. 6d also validated our model (Figs. 6).

Our study showed for the first time that hyperglycemia results in resistance to therapy in pancreatic cancer. However, the mechanism by which high blood glucose results in therapy resistance remained unclear. We observed an enrichment of cancer stem cells that typically contribute towards resistance. Our previous studies show that these cells are also treatment refractory owing to high Aldh1 activity and ABC transporter activity. It is possible that in addition to microbial dysbiosis, the hyperglycemic conditions have direct effect on the tumor tissues and enrich for this population which leads to poor response to chemotherapy [12, 14]. Additionally, hyperglycemia was associated with heavy collagen deposition in the tumors (Fig. 2). The ECM deposition in tumor often prevents efficient drug delivery to the tumors by compressing vasculature. Along with this, we also observed an enrichment of bacterial population that can act in a tumor protective manner. Our analysis microbial metabolome indicated that the microbial dysbiosis was associated with a shift in the metabolome to select for anti-oxidant type metabolites. These typically offer protection against chemotherapy induced oxidative stress, inducing therapy resistance. It is possible that decreased response to therapy was due to complex intra-tumoral, microenvironmental and systemic changes associated with hyperglycemia. At the systemic level, enrichment of tumor protective bacteria and their metabolites that are tumor protective can promote activation of anti-oxidative signaling pathways like NRF2. Further, enrichment of stroma and cancer stem cells in the tumor can also contribute to therapy resistance. However, more experimentation is needed to confirm if all these contributing factors converge from dysbiotic microbiome.

## Conclusion

This study shows that T2D negatively contributes to therapeutic outcomes in pancreatic cancer.

We conclude that enrichment of a “tumor protective” gut bacteria as well as enrichment of a “stem-like” population contributed to this phenomenon. Understanding this better and at a systemic level is likely to help in managing chemoresistance in this disease and improve survival statistics.

## Supplementary information

**Additional file 1: Figure S1.** Validation of T2D model. Animals receiving high fat and streptozotocin do not show change in body weight (A). There is no difference in the tumor take rate in control and T2D group (B).

**Additional file 2: Figure S2.** Quantitation of histology: Ki67 Staining (A) and CD133 staining quantitated in the different tissue sections from the harvested tumors.

**Additional file 3: Figure S3.** Heatmap of pathways enriched in T2D vs Control group.

**Additional file 4: Figure S4.** Metacyc Rendition of the metabolomics pathways identified in C1/D1 and C4/D4.

**Additional file 5: Figure S5.** Metacyc Rendition of metabolomic pathways identified in C4′/D4′.

**Additional file 6: Figure S6.** Significantly changing microbial species in control vs T2D group: Top 50 bacterial genera changed between C1 and D1 (A), C4 vs D4 (B) and after Gem/Pac treatment in C4′ and D4′ group (C).

## Data Availability

The sequence data has been uploaded at Array Express (details in “Methods” section)
