# Supplementary figures and images for: Type 2 diabetes induced microbiome dysbiosis is associated with therapy resistance in pancreatic adenocarcinoma
Source: Microb Cell Fact. 2020 Mar 24;19:75. doi: 10.1186/s12934-020-01330-3 (PMC7092523; doi:10.1186/s12934-020-01330-3)

A.

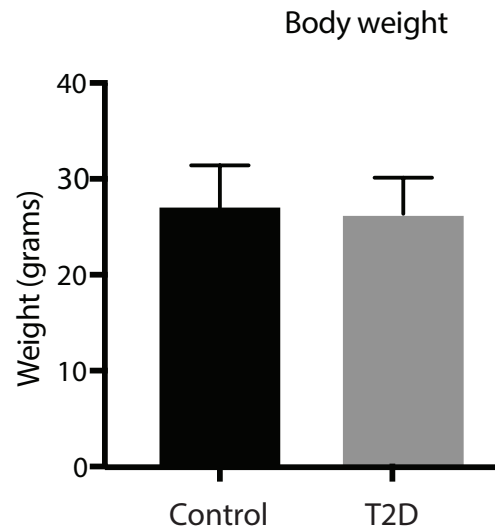

B.

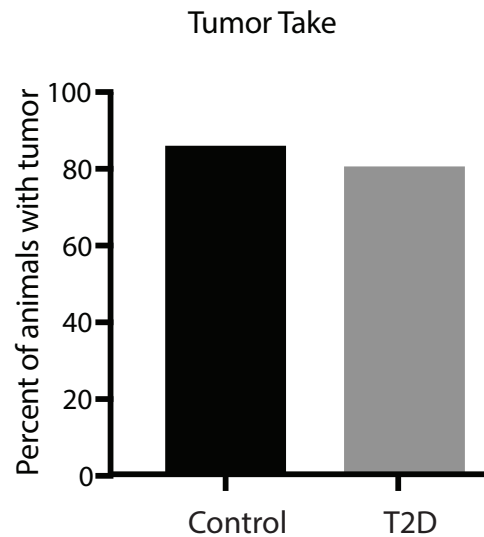

Supplementary Figure 1

Supplement: Supplementary file 1 — Additional file 1: Figure S1. Validation of T2D model. Animals receiving high fat and streptozotocin do not show change in body weight (A). There is no difference in the tumor take rate in control and T2D group (B). [file 12934_2020_1330_MOESM1_ESM.pdf]

A.

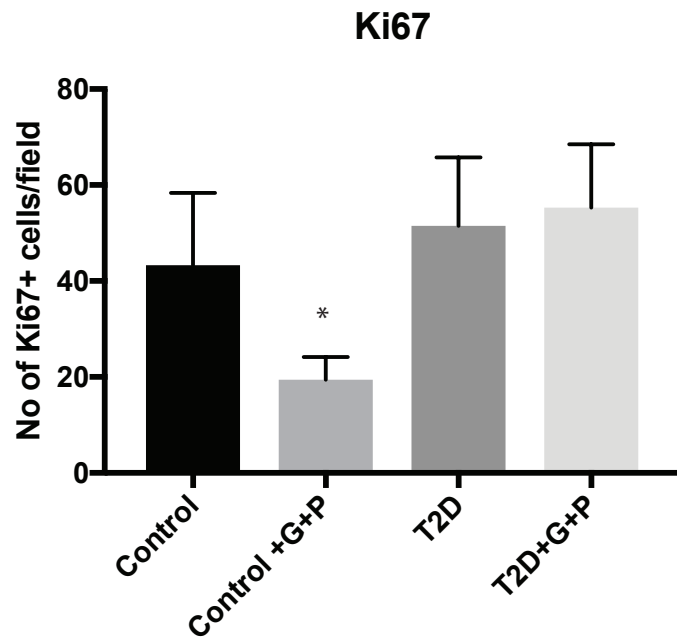

B.

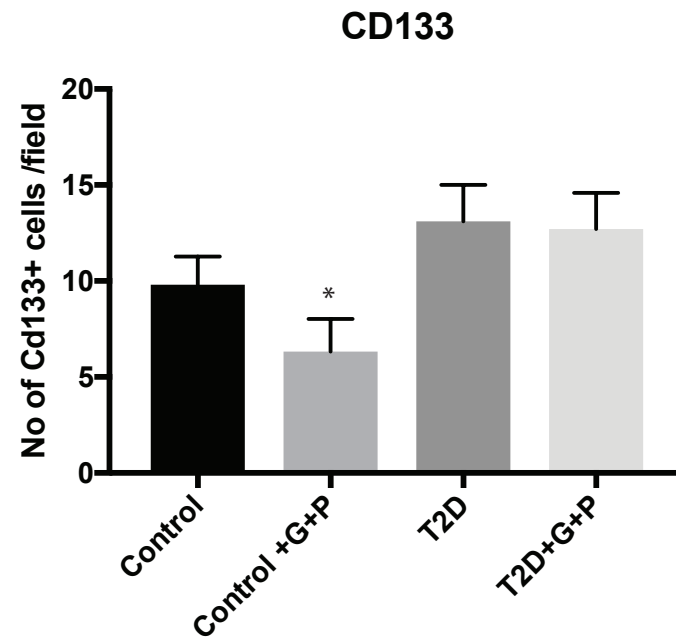

Supplementary Figure 2

Supplement: Supplementary file 2 — Additional file 2: Figure S2. Quantitation of histology: Ki67 Staining (A) and CD133 staining quantitated in the different tissue sections from the harvested tumors. [file 12934_2020_1330_MOESM2_ESM.pdf]

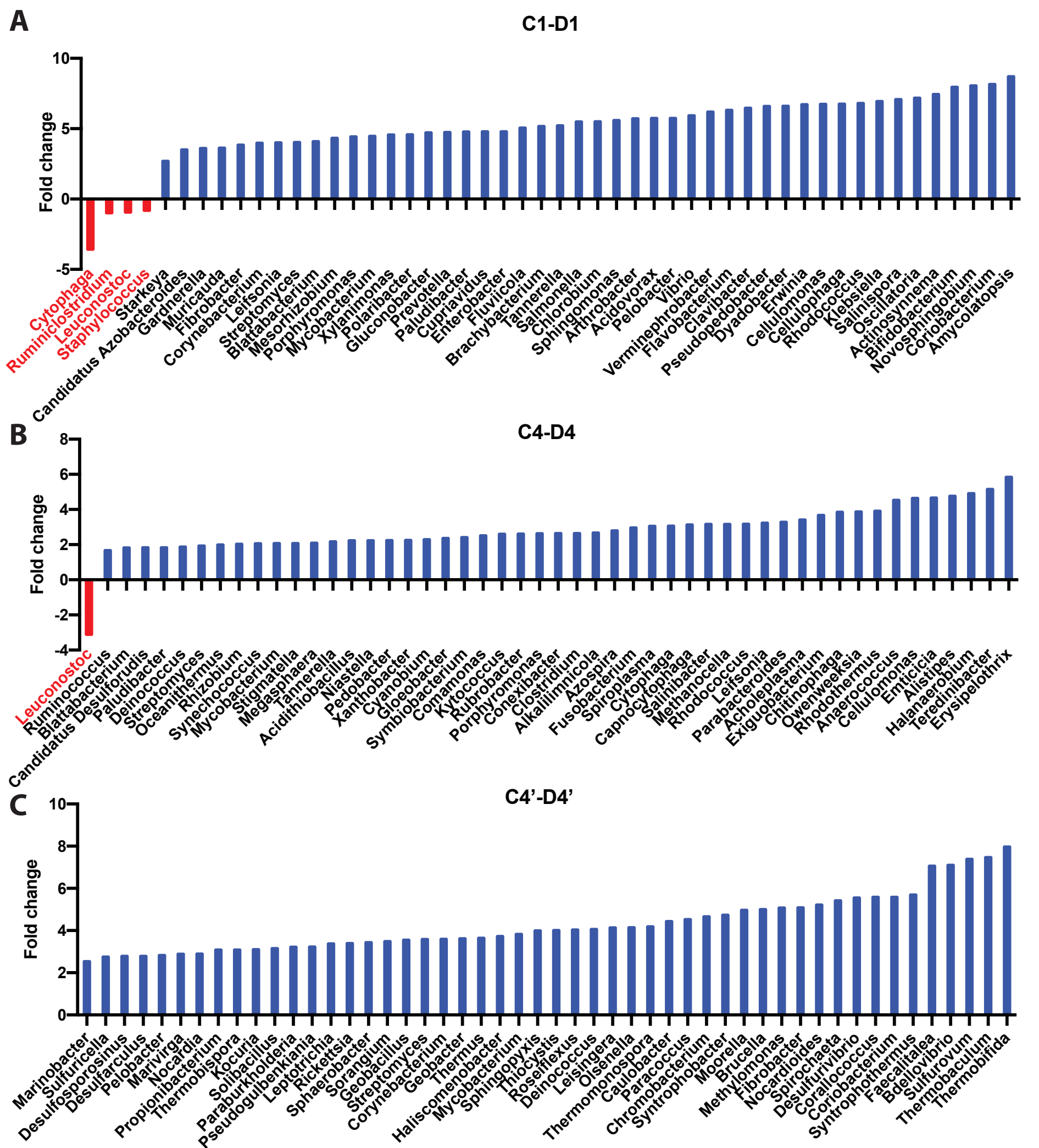

Supplement: Supplementary file 6 — Additional file 6: Figure S6. Significantly changing microbial species in control vs T2D group: Top 50 bacterial genera changed between C1 and D1 (A), C4 vs D4 (B) and after Gem/Pac treatment in C4′ and D4′ group (C). [file 12934_2020_1330_MOESM6_ESM.pdf]
