# Supplementary material for: Type 2 diabetes induced microbiome dysbiosis is associated with therapy resistance in pancreatic adenocarcinoma
Source: Microb Cell Fact. 2020 Mar 24;19:75. doi: 10.1186/s12934-020-01330-3 (PMC7092523; doi:10.1186/s12934-020-01330-3)
Supplement: Supplementary file 3 — Additional file 3: Figure S3. Heatmap of pathways enriched in T2D vs Control group. [file 12934_2020_1330_MOESM3_ESM.pdf]

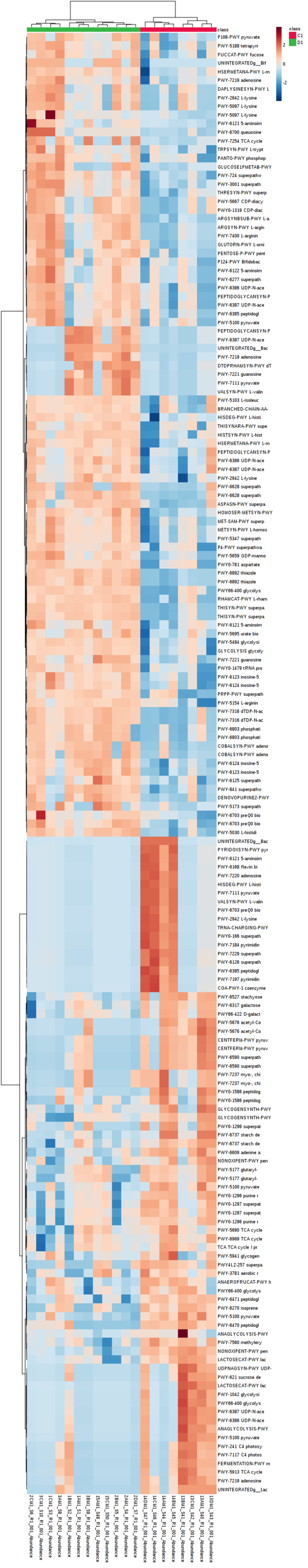

Supplementary Figure 3
