# Supplementary material for: Type 2 diabetes induced microbiome dysbiosis is associated with therapy resistance in pancreatic adenocarcinoma
Source: Microb Cell Fact. 2020 Mar 24;19:75. doi: 10.1186/s12934-020-01330-3 (PMC7092523; doi:10.1186/s12934-020-01330-3)
Supplement: Supplementary file 4 — Additional file 4: Figure S4. Metacyc Rendition of the metabolomics pathways identified in C1/D1 and C4/D4. [file 12934_2020_1330_MOESM4_ESM.pdf]

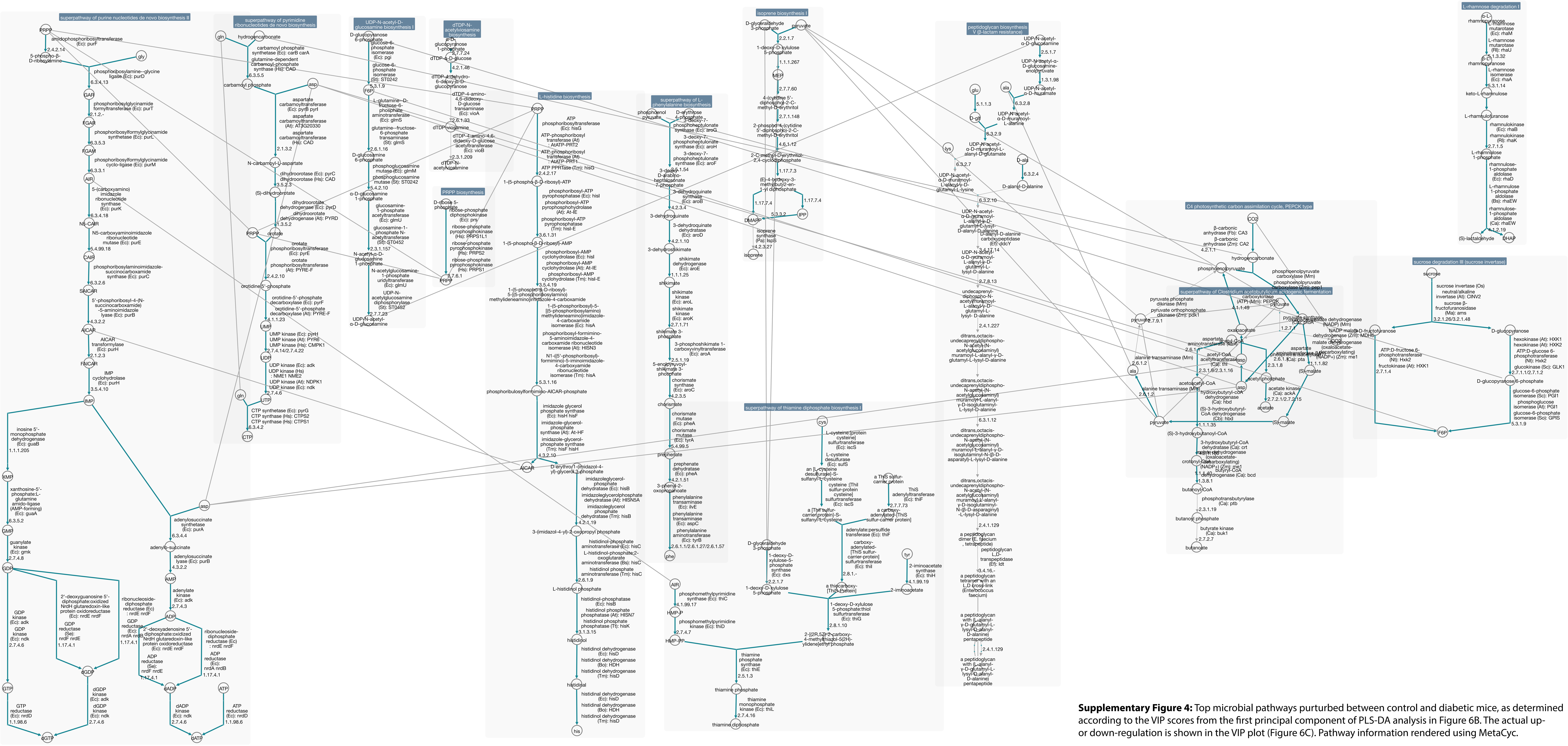

Supplementary Figure 4: Top microbial pathways perturbed between control and diabetic mice, as determined according to the VIP scores from the first principal component of PLS-DA analysis in Figure 6B. The actual up- or down-regulation is shown in the VIP plot (Figure 6C). Pathway information rendered using MetaCyc.

Supplementary Figure 4
