# Supplementary material for: Type 2 diabetes induced microbiome dysbiosis is associated with therapy resistance in pancreatic adenocarcinoma
Source: Microb Cell Fact. 2020 Mar 24;19:75. doi: 10.1186/s12934-020-01330-3 (PMC7092523; doi:10.1186/s12934-020-01330-3)
Supplement: Supplementary file 5 — Additional file 5: Figure S5. Metacyc Rendition of metabolomic pathways identified in C4′/D4′. [file 12934_2020_1330_MOESM5_ESM.pdf]

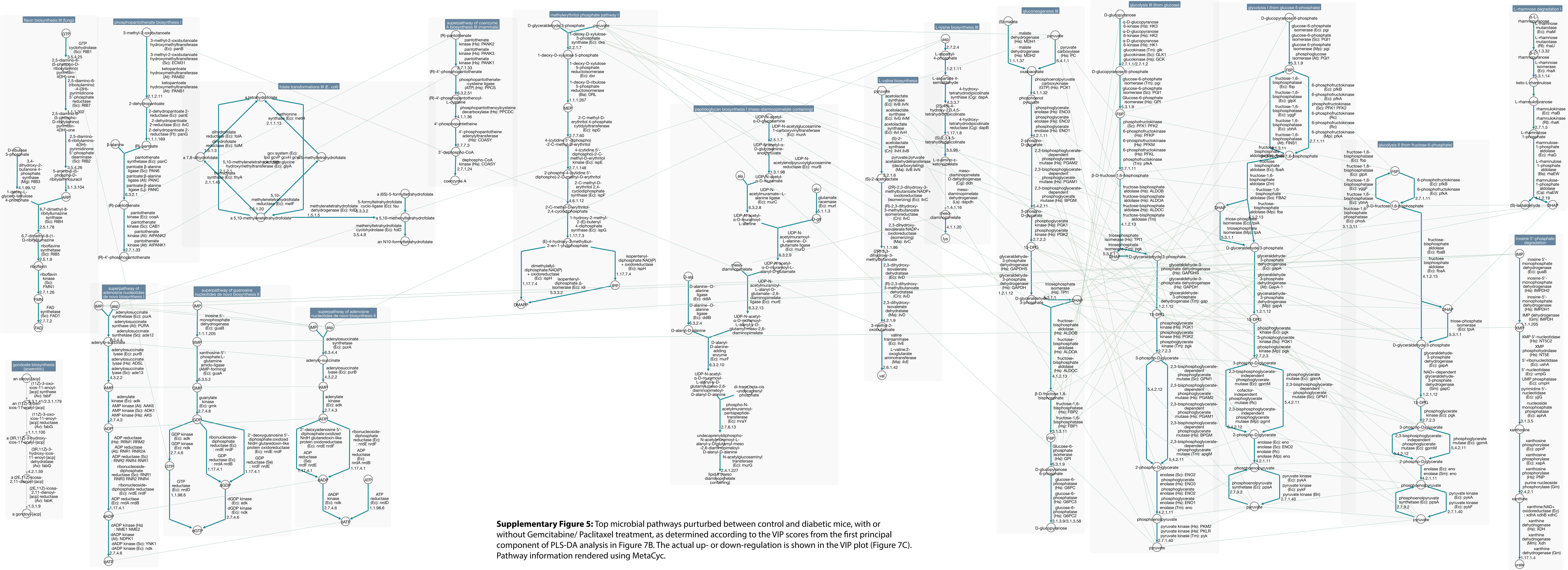

Supplementary Figure 5: Top microbial pathways perturbed between control and diabetic mice, with or without Gemcitabine/ Paclitaxel treatment, as determined according to the VIP scores from the first principal component of PLS-DA analysis in Figure 7B. The actual up- or down-regulation is shown in the VIP plot (Figure 7C). Pathway information rendered using MetaCyc.
